# Hemolytic disease of the fetus and newborn due to Rh(D) incompatibility: A preventable disease that still produces significant morbidity and mortality in children

**DOI:** 10.1371/journal.pone.0235807

**Published:** 2020-07-20

**Authors:** Valeria Pegoraro, Ducciocompet Urbinati, Gerard H. A. Visser, Gian Carlo Di Renzo, Alvin Zipursky, Brie A. Stotler, Steven L. Spitalnik

**Affiliations:** 1 IQVIA Solutions Italy S.r.l., Milan, Italy; 2 Departments of Obstetrics, University Medical Center, Utrecht, the Netherlands; 3 Department of Obstetrics and Gynecology, University of Perugia, Perugia, Italy; 4 Department of Obstetrics and Gynecology, I.M. Sechenov First State University of Moscow, Moscow, Russia; 5 Hospital for Sick Children, Toronto, Ontario, Canada; 6 Department of Pathology and Cell Biology, Columbia University, New York, NY, United States of America; University of New South Wales, AUSTRALIA

## Abstract

In the mid-20^th^ century, Hemolytic Disease of the Fetus and Newborn, caused by maternal alloimmunization to the Rh(D) blood group antigen expressed by fetal red blood cells (i.e., “Rh disease”), was a major cause of fetal and neonatal morbidity and mortality. However, with the regulatory approval, in 1968, of IgG anti-Rh(D) immunoprophylaxis to prevent maternal sensitization, the prospect of eradicating Rh disease was at hand. Indeed, the combination of antenatal and post-partum immunoprophylaxis is ~99% effective at preventing maternal sensitization to Rh(D). To investigate global compliance with this therapeutic intervention, we used an epidemiological approach to estimate the current annual number of pregnancies worldwide involving an Rh(D)-negative mother and an Rh(D)-positive fetus. The annual number of doses of anti-Rh(D) IgG required for successful immunoprophylaxis for these cases was then calculated and compared with an estimate of the annual number of doses of anti-Rh(D) produced and provided worldwide. Our results suggest that ~50% of the women around the world who require this type of immunoprophylaxis do not receive it, presumably due to a lack of awareness, availability, and/or affordability, thereby putting hundreds of thousands of fetuses and neonates at risk for Rh disease each year. The global failure to provide this generally acknowledged standard-of-care to prevent Rh disease, even 50 years after its availability, contributes to an enormous, continuing burden of fetal and neonatal disease and provides a critically important challenge to the international health care system.

## Introduction

Hemolytic Disease of the Fetus and Newborn (HDFN) is caused by maternal alloimmunization to blood group antigens expressed by fetal red blood cells. In severe cases, HDFN induces fetal anemia with increased risks of fetal death, severe neonatal hyperbilirubinemia, and kernicterus [1–3]. Before 1945, ~50% of all fetuses with hemolytic diseases of various etiologies died of kernicterus or hydrops fetalis [4]. Most severe cases of HDFN were attributed to Rh(D) incompatibility between an Rh(D)-negative woman and her Rh(D)-positive fetus, with Rh(D) alloimmunization having occurred during a previous pregnancy [2, 3]. In the 1960s, studies in the United States and in Great Britain determined that passive immunization of Rh(D)-negative mothers with IgG anti-Rh(D), soon after parturition, could protect women from sensitization against Rh(D)-positive red blood cells [5]. This then led to regulatory approval and licensure of IgG anti-Rh(D) preparations for routine post-partum prophylaxis in 1968, more than 50 years ago.

However, in 1977 it was demonstrated that, despite adequate post-natal prophylaxis, ~10% of Rh(D)-negative women continued to develop anti-Rh(D) antibodies, presumably due to small, transplacental, fetal-maternal hemorrhages during pregnancy [6]. To address this issue, antenatal administration of IgG anti-Rh(D) preparations was instituted, which virtually abolished this phenomenon, when combined with standard post-partum prophylaxis [1, 2, 7–9]. Therefore, most current guidelines, prepared by various associations of healthcare professionals involved in preventing and managing HDFN, including obstetricians and gynecologists, pediatricians and neonatologists, hematologists, and specialists in transfusion medicine, recommend that immunoprophylaxis with IgG anti-Rh(D) be given to every non-sensitized Rh(D)-negative woman, as follows: (1) at 28 weeks of gestation during each pregnancy, (2) immediately after delivery of every Rh(D)-positive neonate, and (3) in the context of any other event that could expose her to the Rh(D) antigen (e.g., abortion, miscarriage, abdominal trauma) [3, 4, 7–10]. The only settings in which antenatal anti-D IgG administration is not necessary is when the father is also Rh(D)-negative or if the fetus is successfully typed for Rh(D) status by antenatal cell-free DNA testing using maternal plasma [1, 2].

What has been described up to now is undoubtedly a success story and a great achievement of science and medicine. However, a review of the literature revealed very few publications describing the prevalence of Rh(D) disease in low income countries, where it is not generally considered to be a major problem, presumably due to a lower prevalence of the Rh(D)-negative blood type than in high income countries and an absence of data collection and disease monitoring for this disorder [11–13]. In addition, to our knowledge, no previous studies attempted to demonstrate that both antenatal and post-partum prophylaxis are effectively and consistently used in high income countries. Therefore, the main objective of the present study is to attempt to quantify the worldwide “gap” between the annual number of doses of IgG anti-Rh(D) that should theoretically be administered to minimize the risk of Rh(D) sentitization, and the annual number of doses of IgG anti-Rh(D) that are actually administered.

## Methods

### Parameters and data sources

The parameters needed to calculate the number of doses of IgG anti-Rh(D) that should be administered annually to minimize maternal Rh(D) sensitization are: (1) the number of births per year in each country and (2) the prevalence of the Rh(D)-negative phenotype in those countries.

For most countries, the number of births per year, updated in 2015, was provided by UNICEF [14], which was demonstrated to be a reliable data source in a prior study [13]. For countries for which this information was not available from UNICEF, the number of births per year was calculated based on the number of inhabitants and the birth rates reported in the World Factbook of the Central Intelligence Agency [15].

Most Rh(D)-negative phenotype prevalence estimates were obtained from a prior study [13]; when the relevant information was not included in that study, prevalence estimates were obtained from the study by Flegr [16]. When the Rh(D)-negative prevalence for a given country was not available in either of these two studies, it was calculated as the mean of the Rh(D)-negative prevalence in neighboring countries weighted by the number of inhabitants of each.

The annual number of doses of IgG anti-Rh(D) actually administered in each country was primarily derived from two sources: a prior publication [13] and the IQVIA Multinational Integrated Data Analysis System (MIDAS). MIDAS is the most comprehensive source of information on international drug prices and sales [17]. It summarizes data obtained from IQVIA’s detailed audits of pharmaceutical purchases made by retailers (in 70 countries) and hospitals (in 45 countries). MIDAS contains information on sales of individual products, measured in both currency and physical units, as well as information on the product manufacturer, the active ingredient, brand, form, strength, pack size, and therapeutic class [18]. MIDAS data have been extensively used for research purposes [17–21].

### Analysis

For each country, the following were calculated: (1) the annual number of doses of IgG anti-Rh(D) needed for post-partum immunoprophylaxis and (2) the annual number of doses of IgG anti-Rh(D) needed for antenatal immunoprophylaxis. The number of doses of IgG anti-Rh(D) required for post-partum immunoprophylaxis coincides with the number of Rh(D)-positive neonates delivered by Rh(D)-negative women; for this calculation, it was assumed that all neonates would be typed for the presence or absence of Rh(D). To this end, we calculated the annual number of pregnancies for Rh(D)-negative women by multiplying the annual number of births by the prevalence of the Rh(D)-negative phenotype. Then, we multiplied this number of pregnancies in Rh(D)-negative women by the prevalence of the Rh(D) allele, which represents the probability of having the neonate inherit an Rh(D) allele from the father. The prevalence of the Rh(D) allele was calculated as complementary to the prevalence of the Rh(D)-negative allele; the latter, in turn, due to its recessive inheritance, was calculated as the square root of the prevalence of Rh(D)-negative phenotype. An equivalent methodology has been used previously [11, 13].

The annual number of doses of IgG anti-Rh(D) required for antenatal immunoprophylaxis coincides with the annual number of pregnancies involving an Rh(D)-negative woman and an Rh(D)-positive man; in this case, it was assumed that antenatal prophylaxis would be provided without knowing the Rh(D) status of the fetus. Therefore, the number of doses was calculated by multiplying the number of pregnancies in Rh(D)-negative women by the prevalence of the Rh(D)-positive phenotype. The prevalence of the Rh(D)-positive genotype was calculated as complementary to Rh(D)-negative phenotype prevalence. Nonetheless, there are a few high income countries (e.g., Denmark and the Netherlands) who have national programs to perform antenatal cell-free DNA testing for Rh(D) using maternal plasma [1,2]; in these cases our approach overestimates the number of doses required for antenatal (but not postnatal) immunoprophylaxis.

The annual number of doses of IgG anti-Rh(D) required for post-partum immunoprophylaxis and the sum of the doses of IgG anti-Rh(D) required for both postpartum and antenatal immunoprophylaxis, respectively, set the minimum and optimum annual thresholds for preventing maternal sensitization to the Rh(D) antigen resulting from pregnancy.

To have a global overview regarding the status of immunoprophylaxis required to prevent sensitization to the Rh(D) antigen, the country-level estimates of the annual number of antenatal and post-partum doses of IgG anti-Rh(D) required, and of the annual number of doses of IgG anti-Rh(D) administered, were grouped by Global Burden of Disease (GBD) Super Region. GBD Super Region grouping is based not only on geographic location, but also on a country’s Gross Domestic Product (GDP) [13]. In particular, countries were grouped into the following 7 GBD Super Regions: High Income, Asia East, South East and Pacific, Eastern Europe/Central Asia, North Africa/Middle East, Latin America and Caribbean, Sub-Saharan Africa, and Asia South; S1 Appendix lists the countries included in each GBD Super Region. All GBD Super Region estimates, along with the differences between the number of doses of IgG anti-Rh(D) required for post-partum immunoprophylaxis and the number of doses of IgG anti-Rh(D) administered (i.e., Δ), were calculated. Then, for each GBD Super Region, the number of anti-D IgG doses administered were graphically compared to the minimum and optimum thresholds for preventing maternal sensitization to the Rh(D) antigen. Because data concerning the number of doses actually administered were available for some countries [13, 17], but not for others, we adopted a conservative approach in estimating the number of doses administered worldwide, in an effort to be conservative by minimizing the “gap.” In particular, for countries with data available from both Ref. [13] and [17], we used the source reporting the highest number of doses administered; for countries with no available information, we took the most conservative approach by assuming that the annual number of doses administered actually equaled the annual number of doses required for both antenatal and post-partum immunoprophylaxis.

Finally, to quantify the “gap” in a way that would allow comparisons between countries, we calculated the proportion of the doses of IgG anti-Rh(D) not administered divided by what should have been administered to prevent sensitization of Rh(D)-negative women delivering an Rh(D)-positive baby (i.e., if only post-partum immunoprophylaxis were provided) using the following formula:

$$Gap=\left[1-\frac{anti-Rh(D)\;IgG\;doses\;administered}{anti-Rh(D)\;IgG\;doses\;required\;to\;provide\;post-partum\;immunoprophylaxis}\right]\;X\;100$$

The calculated gaps were then plotted onto a worldwide heat-map. For this analysis, no assumptions were made for countries where information about anti-Rh(D) immunoprophylaxis was not available; these countries are presented in white on the heat-map. As in the GBD Super Region analysis above, for countries where the information about the number of doses of IgG anti-Rh(D) administered was available from both Ref. [13] and [17], the source reporting the highest number of such doses was used to provide the most conservative estimates.

## Results

The annual number of doses of IgG anti-Rh(D) required to provide antenatal and post-partum immunoprophylaxis by GBD Super Region is shown in Table 1, together with the total number of annual doses actually administered and the difference between the number of IgG anti-Rh(D) doses required for post-partum immunoprophylaxis and the number actually administered (i.e., Δ). Focusing on these worldwide estimates, a total of ~13 million annual doses are required globally to prevent sensitization to Rh(D) (i.e., by providing immunoprophylaxis both antenatally and post-partum). However, fewer than 4 million doses are currently administered annually; this outcome does not even achieve the minimum threshold for preventing Rh(D) sensitization by providing only post-partum immunoprophylaxis, which would require more than 5 million doses annually. Finally, more than 2.5 million additional annual doses of IgG anti-Rh(D) are needed outside of the high income countries to achieve post-partum immunoprophylaxis alone (Δ; Table 1).

**Table 1:** The number of anti-Rh(D) doses required annually to prevent maternal sensitization to Rh(D), and the number of anti-Rh(D) doses actually administered annually.

Super GBD Region	Antenatal Doses Needed	Post-partum Doses Needed	Total Doses Needed	Total Doses Administered	Δ
High Income	1,283,343	924,928	2,208,271	1,755,448	-
Asia East, S.East and Pacific	147,939	137,006	284,945	73,640	63,366
Asia South	1,967,810	1,577,724	3,545,534	394,756	1,182,968
Eastern Europe/ Central Asia	616,632	450,982	1,067,614	245,827	205,155
Latin America and Caribbean	896,050	673,265	1,569,315	365,404	307,861
North Africa/Middle East	991,502	751,921	1,743,423	665,683	86,238
Sub-Saharan Africa	1,387,024	1,152,257	2,539,281	110,922	1,041,335
**World**	**7,290,300**	**5,668,083**	**12,958,383**	**3,611,680**	**2,886,923**

**Fig 1:**
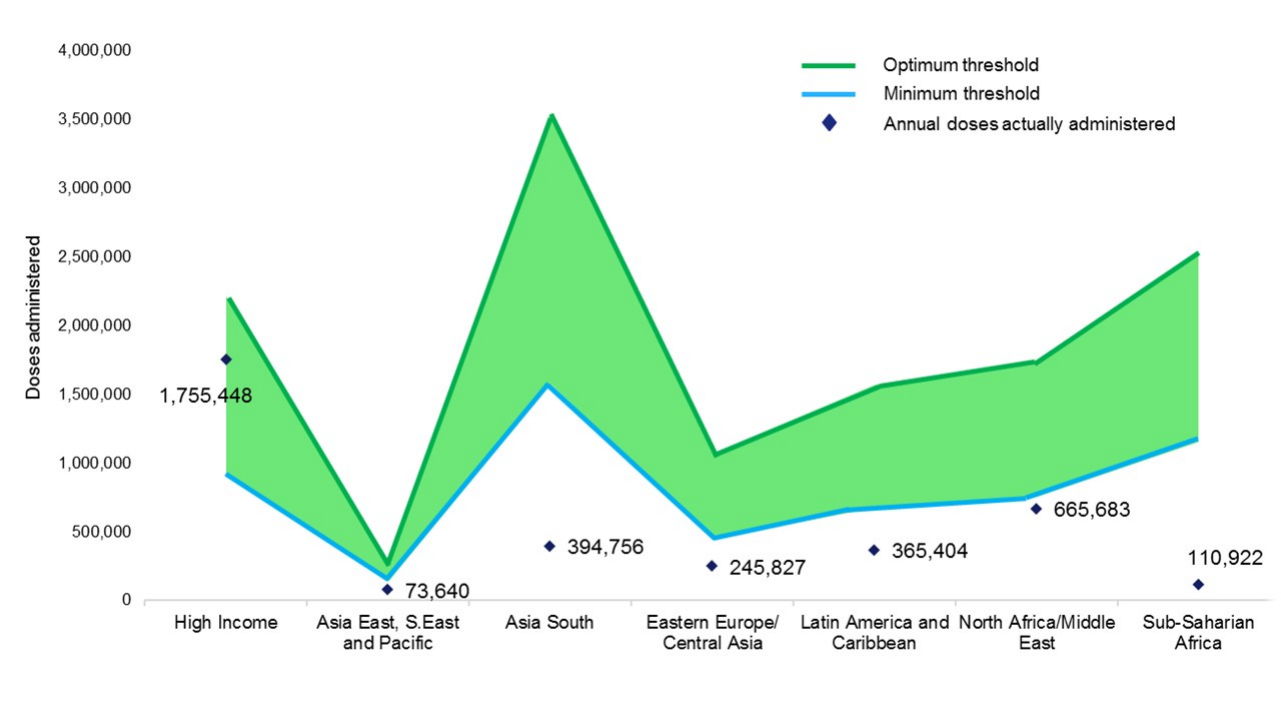
IgG anti-Rh(D) doses actually administered annually, as compared with minimum and optimum thresholds for immunoprophylaxis. The GBD Super Regions are indicated along the x-axis and the annual number of doses of IgG anti-Rh(D) are indicated along the y-axis. The annual numbers of IgG anti-Rh(D) doses actually administered in each GBD Super Region are indicated by filled-in black diamonds. The minimum threshold number of annual IgG anti-Rh(D) doses (i.e., the number required to provide post-partum immunoprophylaxis only) is indicated by the blue line; the optimum threshold number of annual IgG anti-Rh(D) doses (i.e., the number required to provide both antenatal and post-partum immunoprophylaxis only) is indicated by the green line. The “acceptability area” is indicated in light green.

As shown in Fig 1, the “High Income” GBD Super Region is the only one for which the total number of annual doses of IgG anti-Rh(D) administered falls inside the green “acceptability area;” this is, the area between the minimum (i.e., post-partum prophylaxis only) and optimum (i.e., both antepartum and post-partum prophylaxis) thresholds for anti-Rh(D) immunoprophylaxis. For all the other GBD Super Regions, the total number of doses administered falls well below the minimum threshold; Asia South and Sub-Saharan Africa are the GBD Super Regions furthest from the acceptability area (by more than 1 million doses each). The number of doses needed to reach the acceptability area is ~300,000 for the Latin America and Caribbean Super Region and ~200,000 for Eastern Europe/Central Asia Super Region, whereas the North Africa/Middle East, and Asia East, S. East and Pacific Super Regions are ~90,000 and ~60,000 doses below the minimum threshold, respectively.

**Fig 2:**
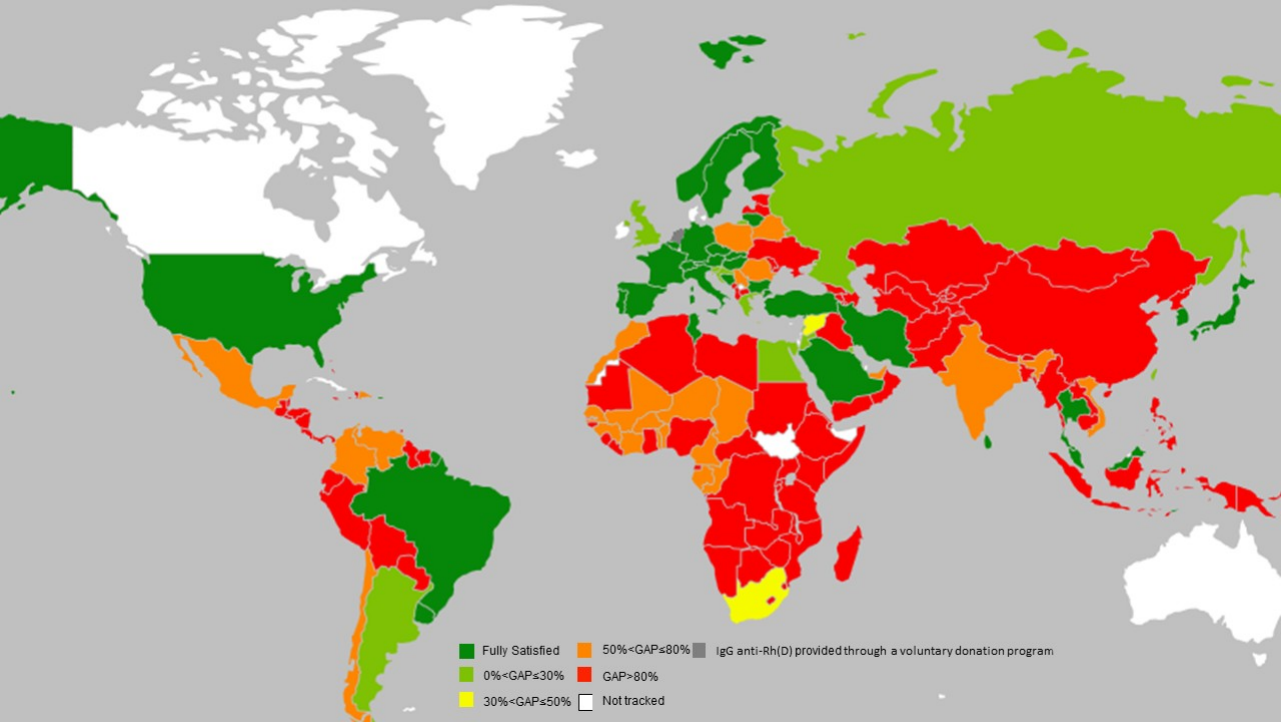
Heat-map representing each country’s gap in providing post-partum Rh(D) immunoprophylaxis. The “gap” is the proportion of annual doses of IgG anti-Rh(D) not administered post-partum divided by those that should have been administered to prevent maternal Rh(D) sensitization. The size of the gap is indicated by various colors; dark green: fully satisfied; light green: gap between 0 and 30%; yellow: gap between 30 and 50%; light orange: gap between 50 and 80%; dark orange: gap greater than 80%; white: data not available because statistics are not tracked in these countries; gray: IgG anti-Rh(D) provided through a national volunteer program [22] and, therefore, not tracked in the MIDAS database.

The heat-map in Fig 2 provides a country-by-country comparison of the proportion of annual IgG anti-Rh(D) doses not administered to those that should have been administered to provide complete post-partum immunoprophylaxis; dark green represents countries with the highest coverage, dark red with the least (S2 Appendix provides a detailed list of each country by the size of the gap and the identify of the GBD Super Region). Although these types of data were available for all countries in the Asia South Super Region, they were not available for 27 countries (indicated in white on the map): 16 of these are High Income countries; 3 each in the Asia East, S.East and Pacific, Latin America and Caribbean, and North Africa/Middle East Super Regions, and one each in the Eastern Europe/Central Asia and Sub-Saharan Super Regions.

In particular, at the extremes, we found:

- 33 countries (17%) for which post-partum immunoprophylaxis is virtually fully satisfied (dark green), of which:
  ○ 15 are High Income countries, representing 43% of that GBD Super Region;
  ○ 6 are Asia East, S.East and Pacific countries, representing 20% of that GBD Super Region;
  ○ 6 are Eastern Europe/Central Asia countries, representing 20% of that GBD Super Region;
  ○ 4 are North Africa/Middle East countries, representing 21% of that GBD Super Region.
  ○ 2 are Latin America and Caribbean countries, representing 6% of that GBD Super Region;
  ○ No countries are in the Asia South and Sub-Saharan GBD Super Regions
- 100 countries (50%) with a gap between post-partum immunoprophylaxis supply and demand higher than 80% (dark red), of which:
  ○ 19 are Asia East, S.East and Pacific countries, representing 63% of that GBD Super Region;
  ○ 5 are Asia South countries, representing 83% of that GBD Super Region;
  ○ 16 are Eastern Europe/Central Asia countries, representing 53% of that GBD Super Region;
  ○ 22 are Latin America and Caribbean countries, representing 71% of that GBD Super Region;
  ○ 5 are North Africa/Middle East countries, representing 26% of that GBD Super Region;
  ○ 33 are Sub-Saharan countries, representing 70% of that GBD Super Region.

## Discussion

Although a method to prevent maternal Rh(D) sensitization in Rh(D)-negative women and, thereby, prevent Rh(D)-disease, was discovered more than 50 years ago, the findings of the current study are tragically surprising and disappointing. They show that (1) worldwide efforts to prevent Rh(D)-negative sensitization are below the minimum threshold and far from the optimum goal, (2) the gap between IgG anti-Rh(D) supply and demand is large in low income countries, and (3) in high income countries, immunoprophylaxis for maternal Rh(D) sensitization also falls below the optimum threshold required to guarantee complete prevention.

Worldwide, the annual “gap” is more than 2.5 million doses below even the minimum threshold for Rh(D) immunoprophylaxis recommended by the WHO [23]. Although previous studies attempted to identify the burden of HDFN due to a lack of immunoprophylaxis in low income countries [11, 13] to our knowledge, this is the first study to estimate the difference between the annual number of doses of IgG anti-Rh(D) required and those administered at a global level. The enormous lack of prevention identified in the current study should be regarded as a global emergency due to a lack of maternal accessibility to IgG anti-Rh(D). As such, cooperation between health authorities, the pharmaceutical industry, and the relevant healthcare providers (e.g., physicians, nurses, midwives) is urgently needed to monitor the ongoing status of this worldwide gap and to guarantee access to, at least, post-partum immunoprophylaxis for all Rh(D)-negative women who deliver an Rh(D)-positive baby.

In addition, no GBD Super Region, including the High Income one, was found to have an acceptable level of immunoprophylaxis. The GBD Super Regions with the highest gap in absolute numbers are Asia South and Sub-Saharan Africa. Consistent with these findings, a prior study reported that Asia South and Sub-Saharan Africa are the two regions with the highest incidence of neonatal death due to kernicterus, of which Rh disease is etiologically relevant [13]. However, it is clear from both the current study and the prior one that the situation is also alarming in other GBD Super Regions.

Another important finding of this study is that, despite the common practice of post-partum immunoprophylaxis in High Income countries, it seems that antenatal immunoprophylaxis is not as widespread as recommended. Previous studies attempting to identify the global burden of Rh(D)-disease due to the failure in preventing Rh(D) sensitization often assumed that Rh(D) disease had been virtually eliminated in High Income countries by IgG anti-Rh(D) immunoprophylaxis given both antenatally and post-partum [3, 4, 7, 9, 12]. However, although the current study estimated that more than 2 million doses of IgG anti-Rh(D) should be administered annually in High Income countries to provide both antenatal and post-partum immunoprophylaxis, we found that only ~1.7 million doses were actually administered annually. Nonetheless, it is possible that this difference is somewhat overestimated, because some High Income countries use non-invasive fetal Rh(D) genotyping programs to limit antenatal immunoprophylaxis to cases where the fetus is genotyped as Rh(D)-positive [24].

The reasons for the continuing burden of Rh disease vary widely, but discussions with representatives from many countries identified some important factors [25]. For example, in Africa, ABO and Rh blood group typing is not routinely performed in many regions, and the cost of IgG anti-Rh(D) may be 4–8 times higher than in high income countries, primarily due to the privatization of pharmacies. In addition, in South America there are often insufficient supplies of IgG anti-Rh(D), whereas in China, IgG anti-Rh(D) immunoglobin is simply not available. In Eastern Europe and Russia, the need for IgG anti-Rh(D) immunoprophylaxis is often forgotten, particularly in the settings of miscarriage, abortion, and abnormal bleeding during pregnancy. Finally, even in High Income countries some patients may not receive optimal care [26] due to inadequate education of health care providers [27, 28].

Nonetheless, the current study has several limitations. First, information about the supply of IgG anti-Rh(D) was not available for all countries. However, for High Income countries, this absence of data does not necessarily reflect a lack of implementation of post-partum immunoprophylaxis. Indeed, for most of these countries, post-partum immunoprophylaxis, at a minimum, is the standard-of-care [1, 24, 26, 29–31]. Because the remaining countries in the High Income GBD Super Region with no available information have relatively low numbers of inhabitants, we hypothesize that their demand for IgG anti-Rh(D) is supplied by neighboring countries. On the other hand, for countries in the other GBD Super Regions for which data were lacking, we believe this could, in itself, reflect poor attention towards immunoprophylaxis. Second, for 22 of the 68 countries for which we used MIDAS data to estimate the total annual number of IgG anti-Rh(D) doses administered, only data from retail distribution channels were available (i.e., for Chile, Estonia, the countries of French West Africa, Greece, Latvia, Lebanon, Morocco, Pakistan, Peru, Tunisia, United Arab Emirates); in addition, for 3 of these 68 countries, only data from hospital channels were available (Portugal, Thailand, Vietnam). Thus, it is possible that the annual number of IgG anti-Rh(D) doses given was underestimated for some of these countries. However, we think that the underestimates are balanced by conservative assumptions made for countries where data were not available. Indeed, for these countries, we assumed that the annual number of IgG anti-Rh(D) doses administered was equal to those needed to provide both antenatal and post-partum immunoprophylaxis. Third, although MIDAS data were updated in 2017 [17], the data in Ref. [13] were from 2013; thus, we cannot exclude that the situation might have changed since 2013 for some countries. Fourth, it is possible that there is some underlying dynamic that cannot be captured by MIDAS data [17]. For example, for the Netherlands, where immunoprophylaxis protocols are effectively in place [2], we found a gap in the administration of post-partum immunoprophylaxis that was higher than 80%. The reason why MIDAS did not track IgG anti-Rh(D) doses in the Netherlands is likely due to the fact that IgG anti-Rh(D) is provided through a voluntary donation program [22] and was not quantifiable using our methods; therefore, we decided to assign the gray color to the Netherlands on the heat-map in Fig 2. Fifth, our estimates of the prevalence of the Rh(D)-negative phenotype in a given country did not take into account the possibility that significant populations of ethnic minorities may have different blood type prevalences, as compared to that of the dominant population. Sixth, even when IgG anti-Rh(D) is available and can be provided, it is possible that the health practioner does not administer it at the appropriate time. Seventh and finally, this analysis did not account for the need for IgG anti-Rh(D) immunoprophylaxis in other obstetrical settings, such as, miscarriage, abortion, ectopic pregnancy, bleeding during pregnancy, external version in the case of breech position, abdominal trauma, amniocentesis, and the double dose of anti-Rh(D) recommended in the setting of Caesarean delivery [3, 7–10, 12, 26]. Nonetheless, we believe that the limitations described above do not markedly affect the overall global analysis and conclusions.

## Conclusion

In summary, the data presented herein identify a global crisis in which hundreds of thousands of Rh(D)-negative women are at risk for becoming sensitized to Rh(D) because of a lack of awareness about, access to, and/or availability of appropriate immunoprophylaxis. This continues to produce a heavy global burden of Rh(D) disease, characterized by fetal demise, severe neonatal anemia, neonatal hyperbilirubinemia, and kernicterus, with hearing loss and cerebral palsy as possible consequences, more than 50 years after the invention of effective measures to prevent this disease.

## Supporting information

S1 Appendix GBD Super Regions. (DOCX)

S2 Appendix Countries identified by the IgG anti-Rh(D) post-partum immunoprophylaxis gap, arranged by GBD Super Region. (DOCX)
