# Supplementary material for: Hemolytic disease of the fetus and newborn due to Rh(D) incompatibility: A preventable disease that still produces significant morbidity and mortality in children
Source: PLoS One. 2020 Jul 20;15(7):e0235807. doi: 10.1371/journal.pone.0235807 (PMC7371205; doi:10.1371/journal.pone.0235807)
Supplement: S1 Appendix — (DOCX) [file pone.0235807.s001.docx]

**S1 Appendix. GBD Super Regions.**

- High Income: Argentina, Austria, Belgium, Chile, Finland, France, Germany, Greece, Italy, Japan, Netherlands, Norway, Portugal, Singapore, Spain, Sweden, Switzerland, United Kingdom, Uruguay, United States, Andorra, Australia, Brunei, Canada, Cyprus, Denmark, Iceland, Ireland, Israel, Liechtenstein, Luxembourg, Malta, Monaco, New Zealand, San Marino
- Asia East, S.East and Pacific: Cambodia, China, North Korea, Fiji, Hong Kong, Indonesia, Kiribati, Laos, Malaysia, Maldives, Marshall Islands, Mauritius, Federated States of Micronesia, Myanmar, Papua New Guinea, Philippines, South Korea, Samoa, Seychelles, Solomon Islands, Sri Lanka, Taiwan, Thailand, Timor-Leste, Tonga, Vanuatu, Vietnam, Nauru, Palau, Tuvalu
- Eastern Europe/ Central Asia: Albania, Armenia, Azerbaijan, Belarus, Bosnia and Herzegovina, Bulgaria, Croatia, Czech Republic, Estonia, Georgia, Hungary, Kazakhstan, Kyrgyzstan, Latvia, Lithuania, Macedonia, Mongolia, Montenegro, Poland, Moldova, Romania, Russia, Serbia, Slovakia, Slovenia, Tajikistan, Turkmenistan, Ukraine, Uzbekistan, Kosovo
- Latin America and Caribbean: Bahamas, Barbados, Belize, Bolivia, Brazil, Costa Rica, El Salvador, Guatemala, Honduras, Nicaragua, Panama, Colombia, Dominica, Dominican Republic, Ecuador, Grenada, Guyana, Haiti, Jamaica, Mexico, Paraguay, Peru, Puerto Rico, Saint Lucia, Saint Vincent and the Grenadines, Suriname, Trinidad and Tobago, Venezuela, Antigua e Barbuda, Cuba, Saint Kitts and Nevis
- Sub-Saharan Africa: Angola, Botswana, Burundi, Cape Verde, Central African Republic, Comoros, Democratic Republic of the Congo, Djibouti, Eq. Guinea, Eritrea, Ethiopia, Benin, Burkina Faso, Cameroon, Chad, Congo, Gabon, Guinea, Côte d'Ivoire, Mali, Niger, Senegal, Togo, Gambia, Ghana, Guinea-Bissau, Kenya, Lesotho, Liberia, Madagascar, Malawi, Mauritania, Mozambique, Namibia, Nigeria, Rwanda, South Africa, Sao Tome and Principe, Sierra Leone, Somalia, Sudan, Swaziland, Uganda, Tanzania, Zambia, Zimbabwe, Sudan del Sud
- Asia South: Afghanistan, Bangladesh, Bhutan, India, Nepal, Pakistan
- North Africa/Middle East: Algeria, Egypt, Iran, Iraq, Jordan, Kuwait, Lebanon, Libya, Morocco, Oman, Saudi Arabia, Syria, Tunisia, Turkey, United Arab Emirates, Yemen, Bahrain, Palestina, Qatar
