# Supplementary material for: Hemolytic disease of the fetus and newborn due to Rh(D) incompatibility: A preventable disease that still produces significant morbidity and mortality in children
Source: PLoS One. 2020 Jul 20;15(7):e0235807. doi: 10.1371/journal.pone.0235807 (PMC7371205; doi:10.1371/journal.pone.0235807)
Supplement: S2 Appendix — (DOCX) [file pone.0235807.s002.docx]

**S2 Appendix. Countries identified by the IgG anti-Rh(D) post-partum immunoprophylaxis gap, arranged by GBD Super Region.**

- Countries with a fully satisfied gap:
  - 15 in the High Income GBD Super Region: Austria, Belgium, Finland, France, Germany, Italy, Japan, Norway, Portugal, Singapore, Spain, Sweden, Switzerland, Uruguay, and the United States;
  - 6 in the Asia East, S.East and Pacific GBD Super Region: Hong Kong, Malaysia, South Korea, Sri Lanka, Thailand and Timor Leste;
  - 6 in the Eastern Europe/Central Asia GBD Super Region: Bosnia and Herzegovina, Bulgaria, Czech Republic, Hungary, Lithuania and Slovakia;
  - 4 in the North Africa/Middle East GBD Super Region: Iran, Saudi Arabia, Tunisia, Turkey;
  - 2 in the Latin America and Caribbean GBD Super Region: Brazil and Puerto Rico;
  - No countries in the Asia South and Sub-Saharan GBD Super Regions.
- Countries with a gap of ≤30%:
  - 3 in the High Income GBD Super Region: Argentina, Greece, and the United Kingdom;
  - 1 in the Asia East, S.East and Pacific GBD Super Region: Taiwan;
  - 3 in the Eastern Europe/Central Asia GBD Super Region: Croatia, Russian Federation, and Slovenia;
  - 4 in the North Africa/Middle East GBD Super Region: Egypt, Jordan, Kuwait, and Lebanon;
  - No countries in the Latin America and Caribbean, Asia South, and Sub-Saharan GBD Super Regions.
- Countries with a gap of 30%-50%:
  - 1 in the North Africa/Middle East GBD Super Region: Syria;
  - 1 in the Sub-Saharan GBD Super Region: South Africa;
  - No countries in the High Icome, Asia East, S.East and Pacific, Latin America and Caribbean, Eastern Europe/Central Asia, and Asia South GBD Super Regions.
- Countries with a gap of 50%-80%:
  - 1 in the High Income GBD Super Region: South Africa;
  - 1 in the Asia East, S.East and Pacific GBD Super Region: Vietnam;
  - 1 in the Asia South GBD Super Region: India;
  - 4 in the Eastern Europe/Central Asia GBD Super Region: Belarus, Poland, Romania, and Serbia;
  - 4 in the Latin America and Caribbean GBD Super Region: Colombia, Dominican Republic, Mexico, Venezuela;
  - 2 in the North Africa/Middle East GBD Super Region: Morocco and United Arab Emirates;
  - 12 in the Sub-Saharan GBD Super Region: Benin, Burkina Faso, Cameroon, Chad, Congo, Gabon, Guinea, Côte d'Ivoire, Mali, Niger, Senegal, Togo.
- Countries with a gap of >80%:
  - 19 in the Asia East, S.East and Pacific GBD Super Region: Cambodia, China, North Korea, Fiji, Indonesia, Kiribati, Lao PDR, Maldives, Marshall Islands, Mauritius, Federated stats of Micronesia, Myanmar, Papua New Guinea, Philippines, Samoa, Seychelles, Solomon Islands, Tonga, Vanuatu;
  - 5 in the Asia South GBD Super Region: Afghanistan, Bangladesh, Bhutan, Nepal, Pakistan;
  - 16 in the Eastern Europe/Central Asia GBD Super Region: Albania, Armenia, Azerbaijan, Estonia, Georgia, Kazakhstan, Kyrgyzstan, Latvia, Macedonia, Mongolia, Montenegro, Moldova, Tajikistan, Turkmenistan, Ukraine, Uzbekistan;
  - 22 in the Latin America and Caribbean GBD Super Region: Bahamas, Barbados, Belize, Bolivia, Costa Rica, El Salvador, Guatemala, Honduras, Nicaragua, Panama, Dominica, Equador, Grenada, Guyana, Haiti, Jamaica, Paraguay, Peru, Saint Lucia, Saint Vincent and the Grenadines, Suriname, Trinidad and Tobago;
  - 5 in the North Africa/Middle East GBD Super Region: Algeria, Iraq, Lybia, Oman, and Yemen;
  - 33 in the Sub-Saharan GBD Super Region: Angola, Botswana, Burundi, Cape Verde, Central African Republic, Comoros, Democrati Republic of the Congo, Djibouti, Equatorial Guinea, Eritrea, Ethiopia, Gambia, Ghana, Guinea-Bissau, Kenya, Lesotho, Liberia, Madagascar, Malawi, Mauritania, Mozambique, Namibia, Nigeria, Rwanda, Sao Tome and Principe, Sierra Leone, Somalia, Sudan, Swaziland, Uganda, Tanzania, Zambia, Zimbabwe.

Finally, there was an apparent gap of >80% in the Netherlands. However, as IgG anti-Rh(D) is virtually universally provided there through a voluntary donation program, we assigned the gray color to the Netherlands on the heat-map in Fig. 2.
